# Sexual and reproductive health aspects in women with polycystic ovary syndrome: An integrative review

**DOI:** 10.18502/ijrm.v20i9.12062

**Published:** 2022-10-10

**Authors:** Mehri Kalhor, Eesa Mohammadi, Shadab Shahali, Leila Amini, Lida Moghaddam-Banaem

**Affiliations:** ^1^Department of Reproductive Health and Midwifery, Faculty of Medical Sciences, Tarbiat Modares University, Tehran, Iran.; ^2^Department of Nursing, Faculty of Medical Sciences, Tarbiat Modares University, Tehran, Iran.; ^3^Nursing Care Research Center (NCRC) and School of Nursing and Midwifery, Iran University of Medical Sciences, Tehran, Iran.

**Keywords:** Polycystic ovary syndrome, Reproductive health, Sexual health, Women.

## Abstract

**Background:**

Polycystic ovary syndrome (PCOS) is one of the most common endocrine disorders in women worldwide, affecting their sexual and reproductive health (SRH).

**Objective:**

This integrative review aimed to identify SRH aspects in women with PCOS by consolidating the findings from previous studies.

**Materials and Methods:**

The present integrative review was conducted through an electronic systematic review search of 1052 manuscripts published from April 2000 to March 2020 using PubMed, SCOPUS, Web of Science, Embase, Google Scholar, MEDLINE, Science Direct, Ovid, and the Cochrane Library. After at least 2 researchers evaluated the articles based on the inclusion and exclusion criteria, 27 papers were accepted. The data were analyzed by thematic analysis.

**Results:**

9 main themes of SRH were obtained: 1) the impact of PCOS-related complications on reproductive health; 2) the lifelong effect of PCOS on reproductive patterns; 3) PCOS and adverse reproductive and pregnancy outcomes; 4) women's need for understanding complications; 5) the financial burden of the disease; 6) women's life experiences and quality of life; 7) sexual disorders; 8) psychological concerns and issues; and 9) femininity feelings and roles.

**Conclusion:**

We were able to identify and categorize various aspects of SRH needs for women with PCOS. These categories can facilitate a more comprehensive assessment of SRH, including previously neglected areas. We suggest that these aspects should be considered in the health plans of women with PCOS.

## 1. Introduction 

One of the key aspects of women's lives is sexual and reproductive health (SRH). According to the World Health Organization, reproductive health is defined as the Status of physical, mental and social well-being of the reproductive system (1). Reproductive health care includes lifelong health (from birth to death), and aims to help individuals and families improve health, empower women, and increase health care access (2, 3). SRH covers a wide range of care, including for maternal and newborn health, family planning, sexually transmitted infections (STIs) and HIV, reproductive system malignancies, infertility diagnosis and treatment, as well as reproductive health education, and male participation (4).

Today, the health and well-being of women, who consist half of the population, is considered as a human right, which has also an outstanding impact on health of both the family and society. However, health rights, particularly women's SRH rights, are not yet fully recognized in many parts of the world. According to published statistics on the global burden of diseases in 1996, 22% of lives lost in women of childbearing age were due to neglect of SRH and related health problems such as unplanned pregnancy, STIs, and AIDS (5).

All over the world, in women of reproductive age, polycystic ovary syndrome (PCOS) is the most common endocrine disorder, which significantly affects reproductive health. Its prevalence in different regions varies between 2.2-26%. The syndrome is a combination of at least 2 of 3 conditions, including hyperandrogenism, chronic anovulation, and polycystic ovaries. It is often associated with insulin resistance and obesity (6). PCOS may affect the reproductive, metabolic, and endocrine systems, leading to menstrual disorders, endometrial hyperplasia, abnormal uterine bleeding, oligo-ovulation, infertility, and a significant reduction in quality of life. Identification of SRH aspects is required to create a comprehensive and effective health plan (7). Although some studies have evaluated some aspects of SRH such as infertility, sexual disorders, pregnancy complications, and quality of life for women with PCOS (8-10), no comprehensive study has evaluated all SRH aspects in women with PCOS.

In this article, we present an integrative review to identify SRH aspects in women with PCOS, as integrative review is a unique method that summarizes a variety of past theoretical and clinical studies to provide a more extensive understanding of a particular phenomenon or a health problem and a variety of relevant perspectives (11). This method emphasizes the emergence of new insights through a combination of evidence from studies conducted using a wide range of research methods.

## 2. Materials and Methods

The present study is an integrative review that assesses the SRH aspects of women with PCOS based on English and Persian language peer-reviewed publications and grey literature published from April 2000 to March 2020.

Since no comprehensive study of all different domains of SRH in women with PCOS was found, the authors conducted a comprehensive literature search with different key words related to each domain of SRH in these women to answer the main question of this study: what are the main aspects of SRH in women with PCOS?

Whittemore and Knafl have stated the 5 stages of an integrative review as: problem identification, literature search, data evaluation, data analysis, and presentation of findings; the authors used their method for this study (11). In this method, rigor of the studies, and relevance of data are scaled as either high, or low (a 2-point scale).

We used PRISMA flow diagram to perform the study steps. The 5 steps of integrated reviews were also used (12). So that we adapted the 5 stages of the integrative review study with the stages of the PRISMA flow diagram for systematic reviews.

### Identification

The search terms consisted of several key words for a comprehensive search, including various forms of `polycystic ovary syndrome' and various forms of `SRH'. For example, the search strategy used in the Medline database is listed below:

(“sexual health"[MeSH Terms] OR [“sexual" [All Fields] AND “health" [All Fields]] OR “sexual health" [All Fields] OR [“reproductive health" [MeSH Terms] OR (“reproductive" [All Fields] AND “health" [All Fields]] OR “reproductive health" [All Fields]] OR [“sexual behavior" [MeSH Terms] OR [“sexual" [All Fields] AND “behavior" [All Fields]] OR “sexual behavior" [All Fields] OR “sexual" [All Fields] OR “sexually" [All Fields] OR “sexualities" [All Fields] OR “sexuality" [MeSH Terms] OR “sexuality" [All Fields] OR “sexualization" [All Fields] OR “sexualize" [All Fields] OR “sexualized" [All Fields] OR “sexualizing" [All Fields] OR “sexuals" [All Fields]] AND [“reproductive health" [MeSH Terms] OR [“reproductive" [All Fields] AND “health" [All Fields]] OR “reproductive health" [All Fields]] AND [“woman" [All Fields] OR “women" [MeSH Terms] OR “women" [All Fields] OR “woman" [All Fields] OR “women s" [All Fields] OR “women" [All Fields]] OR [“female" [All Fields] OR “female" [MeSH Terms] OR “female" [All Fields] OR “females" [All Fields] OR “female s" [All Fields] OR “females" [All Fields]] AND “need" [All Fields] AND [“polycystic ovary syndrome" [MeSH Terms] OR [“polycystic" [All Fields] AND “ovary" [All Fields] AND “syndrome" [All Fields]] OR “polycystic ovary syndrome" [All Fields] OR [“polycystic" [All Fields] AND “ovarian" [All Fields] AND “syndrome" [All Fields]] OR “polycystic ovarian syndrome" [All Fields]].

The following databases were searched: Elsevier, Guideline Central, Johns Hopkins Medicine.org, Springer, Ovid, MEDLINE, Science Direct, ProQuest, Scopus, EBSCO host, Cochrane Library, PubMed and Google Scholar; and Iranian databases including SID (Scientific information database), Magiran, Irandoc, and Iran Medex.

The inclusion criteria were: research articles published since 2000; on women with PCOS; discussing SRH needs; in Persian or English; with full text available; and containing guidelines related to the needs, problems, and complications of the disease. Studies examining other conditions or groups were excluded.

### Screening

**Figure 1:**
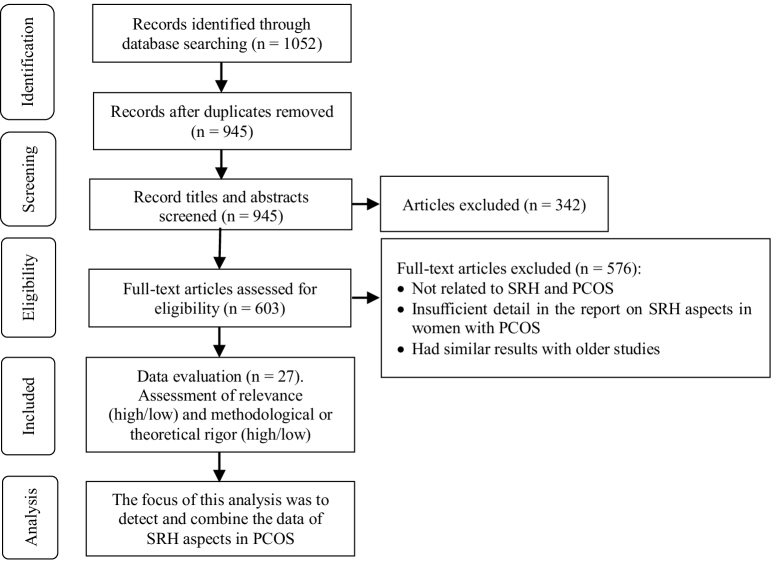
Flow chart of integrative review method.

1052 articles were imported into the Endnote software (EndNote X9, Clarivate, Philadelphia, PA, USA (13) and duplicates were removed. 2 reviewers independently screened the titles, abstracts, and keywords, as well as the year of the study to determine whether they were relevant. Subsequently, the full text of each of the 603 remaining articles was reviewed. Any dispute was settled by discussion and agreement or, if necessary, in consultation with a third reviewer. All the reasons for the exclusion of full text articles were documented (e.g., focusing only on treatment, pathologic basis of PCOS, not related to SRH aspects in PCOS). The final 27 remaining articles included reviews, qualitative studies, quantitative studies, and combined methods studies on PCOS, with a focus on the SRH aspects of PCOS. Two researchers independently assessed the articles using the mixed methods appraisal tool (MMAT) (14), and the assessing the methodological quality of systematic reviews tool (AMSTAR) (15). In case of disagreement, a third researcher reviewed the article. This process is displayed in a PRISMA flow chart (12) (Figure 1).

MMAT is designed for the systematic assessment of mixed studies, i.e., reviews that include qualitative, quantitative, and mixed methods studies. It helps reviewers assess the methodological quality of 5 types of studies: qualitative studies, randomized controlled trials, non-randomized studies, quantitative descriptive studies, and mixed methods studies. MMAT cannot be used to assess the quality of non-empirical papers such as reviews and theoretical papers. Some particular research such as economic and diagnostic accuracy studies also cannot be assessed with MMAT. This tool comprises of 2 parts: checklist (Part I) and explanation of the criteria (Part II).

In the checklist, depending on the type of study and the methodological quality criteria, the evaluator answers `yes', `no' or `I cannot say'. Scoring based on answers is categorized as 0%, 25%, 50-75%, or 100%, and higher scores indicate higher quality (16).

AMSTAR is a commonly used tool for assessing the methodological quality of randomized trials in systematic reviews. The instrument is an 11-item questionnaire that asks reviewers to answer `yes', `no', `can't answer' or `not applicable' with the scoring as: low quality (score 0-4); medium quality (score 5-8); and high quality (score 9-11) (17).

### Data evaluation

The quality of the articles that did not have the routine format of questionnaires was evaluated by the research team in terms of the relationship between the content of the article and the purpose of the research, the type of journal, and the sources of the article. Weak, medium, and strong were the evaluation results.

### Ethics considerations

This study received approval from the Ethics Committee of Tarbiat Modares University, Tehran, Iran (Code: 96-07-03, 4275).

### Statistical analysis 

The compilation of tables and comparative data analysis were conducted based on Whittemore and Knafl's suggestions (11). Qualitative thematic analysis was performed to develop themes from the categories and the repetitive patterns of the included articles (18). The analysis focused on identifying and categorizing SRH data of women with PCOS. A code framework was created to identify data patterns and separate distinct components. The codes were then divided into potential themes and examined to ensure that the overall code framework contributed to the study's purpose and content of the dataset. Prevalent themes were identified and analyzed based on linkages of concepts (18). The research team members also discussed their ideas and interpretations during the process.

## 3. Results

All 27 selected articles were evaluated and scored by the research team according to the MMAT and AMSTAR criteria (Table I). None of the 27 selected articles were of low quality and therefore all were included in the synthesis and analysis stages. The final articles (n = 27) consisted of a variety of quantitative (n = 8), qualitative (n = 7), systematic review (n = 11), and mixed method (n = 1) studies. As the study aim was to discover and consolidate knowledge rather than examining the evidence of impact, no articles were excluded just because of the study method.

**Table 1:** Evaluation and scoring of the quality of the 27 selected articles with the MMAT and AMSTAR tools


**Author, yr (Ref)**	**Method**	**Participants**	**MMAT or AMSTAR scores**
**Teede ** ***et al*** **., 2010 (19) **	Review	- 100%
**Roos ** ***et al*** **., 2011 (20) **	Retrospective cohort	Swedish women, including 3787 births in women with PCOS and 1, 191, 336 births in women without PCOS	100%
**Fauser ** ***et al.*** **, 2012 (21)**	Consensus	- There was no routine format for questionnaire evaluation. The research team assessed quality in terms of the relationship between the content of the article and the purpose of the research, the type of journal, and the sources of the article. Good
**Li ** ***et al*** **., 2018 (22)**	Retrospective cohort	3 groups were included: group A included 6000 healthy women, group B included 24,566 women with PCOS without pre-treatment, and group C included 222 patients with PCOS with treatment	100%
**Sánchez-Ferrer ** ***et al*** **.,** ** 2020 (23) **	Case-control study	117 women with PCOS and 153 controls	100%
** Azziz ***et al*** **., 2005**** ** (24)**	Systematic review	- -No mention of grey literature -No mention of the characteristics of the included studies -No mention of the scientific quality of the assessed studies 75%
** Pasquali ***et al*** **., 2005**** ** (25)**	Review	- There was no routine format for questionnaire evaluation. Quality was assessed by the research team in terms of the relationship between the content of the article and the purpose of the research, the type of journal, and the sources of the article. Good
** Pfister and Rømer,** ** 2017 (26)**	In-depth individual and semi-structured interviews	21 Danish women with PCOS	100%
** Rotterdam ESRHE/** ** ASRM sponsored** ** PCOS consensus** ** workshop group 2004** ** (27)**	Report and consensus	- There was no routine format for questionnaire evaluation. Quality was assessed by the research team in terms of the relationship between the content of the article and the purpose of the research, the type of journal, and the sources of the article. Medium
** Norman ***et al*** **., 2002**** ** (28)**	Review	- There was no routine format for questionnaire evaluation. Quality was assessed by the research team in terms of the relationship between the content of the article and the purpose of the research, the type of journal, and the sources of the article. Good
** Bellver ***et al*** **., 2018**** ** (6)**	Review	- -No mention about grey literature -No mention about the characteristics of the included studies -No mention of the scientific quality of the included studies 75%
** Rzońca ***et al*** **., 2018**** ** (29)**	Cross-sectional, correlational	504 women with PCOS	100%
** Zare Mobini ***et al*** **.,**** ** 2018 (30)**	Exploratory mixed methods, consisted of 3 consecutive phases	-A phase in women with PCOS -Another phase with 2 groups of women with and without PCOS	100%
** Amiri ***et al*** **., 2014 (31)****	Qualitative study with semi-structured interviews	23 women with PCOS	100%
** Jalilian ***et al*** **., 2015**** ** (32)**	Meta-analysis review	19,226 women from 10-45 yr	81% No mention of publication bias assessed and methodological rigor
** Eftekhar ***et al*** **., 2014**** ** (33)**	Cross-sectional study	130 married women with PCOS	80% The sampling strategy was not clear
** Bazarganipour ***et al*** **.,**** ** 2014 (8)**	Cross-sectional study	300 women with PCOS	80% Risk of nonresponse bias was not clear
** Allahbadia and** ** Merchant, 2011 (34)**	Systematic review	- There was no routine format for questionnaire evaluation. Quality was assessed by the research team in terms of the relationship between the content of the article and the purpose of the research, the type of journal, and the sources of the article. Good
** Mahmouda ***et al*** **.,**** ** 2015 (35)**	Cohort study	180 women with PCOS divided into 2 groups: Overweight/obese (BMI > 25) and healthy-weight women (BMI < 25)	80% The sample size was too small
** Weiss and Bulmer,** ** 2011 (36)**	Mixed methods study with phenomenology method	12 women with PCOS aged 18-23 yr	100%
** Kaczmarek ***et al*** **.,**** ** 2016 (37)**	Systematic review-meta-analysis	English and French language articles focusing on HRQoL in patients with PCOS aged 13-24 yr	100%
** Hashemi ***et al*** **., 2014**** ** (38)**	Cross-sectional study	591 married women with PCOS aged 18-45 yr	80% No mention of the risk of nonresponse bias
** Holton ***et al*** **., 2018 (39)****	Deep qualitative interviews with women with PCOS	70 women of reproductive age with PCOS	80% The qualitative data collection methods were not adequate to address the research question
** Avery and** ** Braunack-Mayer,** ** 2007 (40)**	Deep qualitative interviews with women with PCOS	10 South Australian women with PCOS	100%
** Sanchez and Jones,** ** 2016 (41)**	Review magazines related to women	Well-known women's magazine data	90% Not a known evaluation tool for assessing selected articles
** Mazloomy** ** Mahmoodabad, ***et al*** **.,**** ** 2016 (42)**	Cross-sectional descriptive study	70 women with PCOS	80% The sample size was too small
** Nasiri Amiri ***et al*** **.,**** ** 2013 (43)**	Content analysis	20 women aged 18-39 yr with PCOS	100%
MMAT: Mixed methods appraisal tool, AMSTAR: Assessing the methodological quality of systematic reviews, PCOS: Polycystic ovary syndrome, BMI: Body mass index			

Initial codes were created based on the data extracted from the 27 articles. The research team then categorized these initial codes and consolidated similar codes. Ultimately, 9 themes were derived related to SRH aspects in women with PCOS. The identified themes were: (1) the impact of PCOS-related complications on reproductive health; (2) the lifelong effects of PCOS on reproductive patterns; (3) PCOS and adverse reproductive and pregnancy outcomes; (4) women's need for understanding complications; (5) the financial burden of the disease; (6) women's life experiences and quality of life; (7) sexual disorders; (8) psychological concerns and issues; and (9) femininity feelings and roles. Each theme is explained below. Table II lists the extracted themes from the selected articles, and the aims of the studies that investigated these themes.

**Table 2:** Extracted themes from the selected articles.


**Author, yr (Ref)**	**Themes**
**Pasquali ** ***et al.*** **, 2006 (25) **	
**Rotterdam ESHRE/ASRM, 2004 (27) **	
**Norman ** *** et al.*** **, 2002 (28) **	The effect that complications of PCOS have on the reproductive health of women with PCOS
**Jalilian ** *** et al.*** **, 2015 (32) **	
**Allahbadia and Merchant, 2011 (34)**	
**Bellver ** ***et al.*** **, 2018 (6) **	The effect of PCOS on the changing reproductive health patterns throughout
**Fauser ** ***et al.*** **, 2012 (21) **	the life stages from embryonic to old age
**Roos ** ***et al.*** **, 2011 (20) **	
**Li ** ***et al.*** **, 2018 (22) **	
**Mahmoud ** ***et al.*** **, 2015 (35) **	The effect of PCOS on pregnancy, childbirth, and neonatal outcomes
**Holton ** ***et al.*** **, 2018 (39) **		
**Avery and Braunack-Mayer, 2007 (40) **		The fertility-related information needs and preferences of women with PCOS **Teede ** ***et al.*** **, 2010 (19) **	
**Azziz ** ***et al.*** **, 2005 (24) **	The financial burden of the reproductive complications resulting from PCOS	
**Amiri ** ***et al.*** **, 2014 (31) **		
**Kaczmarek ** ***et al.*** **, 2016 (37) **	
**Mazloomy Mahmoodabad ** ***et al.*** **, 2012 (42) **	
**Rzońca** *** et al.*** **, 2018 (29)**	The impact of the reproductive complications of PCOS on quality of life
**Bazarganipour ** ***et al.*** **, 2014 (8) **		
**Eftekhar ** ***et al.*** **, 2014 (33) **		
**Hashemi ** ***et al.*** **, 2014 (38) **		The effect of PCOS on sexual function
**Zare Mobini ** ***et al.*** **, 2018 (30) **		
**Sanchez and Jones, 2016 (41) **	The effect of reproductive complications of PCOS on the concerns and	
**Nasiri Amiri ** ***et al.*** **, 2013 (43) **	psychological health of women with PCOS
**Weiss and Bulmer, 2011 (36)**	
**Sánchez-Ferrer ** ***et al.*** **, 2020 (23) **	
**Pfister and Rømer, 2017 (26) **	The effect of PCOS on the role of femininity
PCOS: Polycystic ovary syndrome	

### Theme 1: The effect that complications of PCOS have on the reproductive health of women with PCOS

This theme examines the impact of PCOS-related complications on affected women's reproductive health. These complications include hirsutism, hyperandrogenism, anovulation, acne, alopecia, menstrual disorders, cardiovascular diseases, weight gain, insulin resistance, diabetes, obesity, and infertility (25, 27, 28). In a study conducted in Iran, the prevalence of hirsutism was 13%, acne 26%, cosmetic problems (including acne and hirsutism) 9%, menstrual disorders 28%, overweight 21%, obesity 19% and infertility 8% (32). The effects of PCOS are reflected in its harmful effects on the body's physiology and metabolism and its potential long-term consequences, such as multisystem disorders including obesity, abnormal gonadotropin function, androgen overproduction, and insulin resistance (34).

Obesity-induced hyperandrogenism in women with PCOS leads to delayed menarche, ovulation dysfunction, and infertility. Ovarian dysfunction usually presents as oligomenorrhea/amenorrhea due to oligo-ovulation/chronic anovulation. Prolonged ovulation can lead to dysfunctional uterine bleeding. Most women with PCOS have ovarian dysfunction, with 70-80% having oligomenorrhea or amenorrhea. Menorrhagia can occur with hyperestrogenism and endometrial hyperplasia, which is exacerbated by increased estrogen levels in obesity (25).

PCOS is the most common cause of infertility due to anovulation. This complication accounts for 90-95% of the reasons that women go to infertility clinics. However, 60% of women with PCOS are fertile (defined as the ability to conceive within 12 months), although it often takes a long time for these women to get pregnant. 90% of people with PCOS and infertility are overweight. Obesity independently exacerbates infertility, reduces the effectiveness of infertility treatment, and increases the risk of miscarriage (25). Therefore, due to reduced pregnancy success and increased adverse events in pregnancy in overweight women, body mass index should also be considered in assisted reproductive therapies (25, 28).

### Theme 2: The effect of PCOS on the changing reproductive health patterns throughout the life stages from fetal period to old age 

Theme 2 discusses the impact of lifelong complications arising from adolescence to adulthood and old age, and the differences in disease presentation in different age groups. Significant complications of the disease during adolescence include hirsutism, acne, and menstrual disorders. During reproductive ages, PCOS may cause infertility, menstrual disorders, and pregnancy complications. Also in this period, PCOS can affect the use of certain types of contraception and women's quality of life. In old ages, long-term metabolic and cardiovascular complications might occur which ultimately increase the risk of cancers (21). In women of different ages, the risk of endometrial cancer, cardiovascular diseases, contraception issues, menstrual disorders, hirsutism, and acne should be considered. Therefore, paying attention to the clinical symptoms of PCOS in different age groups from adolescence through menopause is important for accurate and rapid diagnosis (6).

### Theme 3: The effect of PCOS on pregnancy, childbirth, and neonatal outcomes

Theme 3 shows the association of PCOS with many reproductive complications such as menstrual irregularities, hyperandrogenism, obesity, insulin resistance, and elevated serum luteinizing hormone levels, and also with adverse pregnancy outcomes, such as preeclampsia, preterm delivery, and gestational diabetes. Neonates born from mothers with PCOS are more likely to be large for gestational age, and have an increased risk of meconium aspiration and low Apgar scores ( < 7 at 5 min after birth). Therefore, women with PCOS need more care during pregnancy and childbirth (20). The prevalence of gestational diabetes, induced hypertension, and preterm delivery is higher in pregnant women with PCOS who are not treated appropriately (22). Also, women with PCOS who are overweight or obese are older at menarche and have more abortions and menstrual disorders (35).

### Theme 4: The fertility-related information needs and preferences of women with PCOS

Theme 4 addresses the fertility-related information needs and preferences of women with PCOS. Previous studies have shown that women with PCOS are concerned about the effect of PCOS on the birth of a baby and ask questions about fertility consequences such as whether they can become pregnant, how to prepare for pregnancy, and what they should do before trying to conceive. Therefore, these women are looking for extensive information about fertility and PCOS, because these issues are their priorities. However, views differ on the most useful sources of information on fertility for women. Women use modern means such as the internet, electronic books, brochures, pamphlets and medical applications to obtain information. With enough information about the disease, women with PCOS can decide on treatment strategies and choose their health priorities because they have enough information to participate in a joint decision with their treatment team about having a baby and achieving their fertility goals (40). Prenatal care, including counseling and information on appropriate interventions and self-management strategies to optimize health and improve the chances of pregnancy, may help women with PCOS (39).

### Theme 5: The financial burden of the reproductive complications resulting from PCOS

Theme 5 focuses on the financial burden caused by the reproductive effects of PCOS in women with this condition. A review study found that the financial burden of the disease in women with PCOS from the USA, Greece, and UK includes $1.35 million per year for menstrual irregularities and abnormal uterine bleeding (31.0% of the total), $533 million for infertility care (12.2% of the total), $77.1 million for diabetes (40.5% of the total), and $622 million for the treatment of hirsutism (14.2% of the total). In the initial assessment of the study, the average annual financial burden of the disease was $93 million (2.1% of total annual health budget), and the study authors concluded that the total value of the assessment and provision of care for women of childbearing age with PCOS was $4.36 billion (24).

An Australian study also found that the estimated economic burden of PCOS in Australia in 2010 was $400 million (31% related to menstrual dysfunction, 12% to infertility, and 40% to PCOS-related diabetes), which represents a major health problem and a high economic burden. For fertility, the estimated cost per birth is quite high for Australian women with PCOS who are overweight and infertile ( 275,000 /baby) (19).

### Theme 6: The impact of the reproductive complications of PCOS on quality of life

Theme 6 discusses the impact of disease complications on the affected women's quality of life, and how improvement in fertility outcomes can lead to improved quality of life. Data contributing to this theme indicate that PCOS can lead to various fertility-related complications, including physical impacts (obesity, hirsutism, hair loss, acne, menstrual disorders, infertility, ovarian cysts), mental impacts (depression, hopelessness, fear, and anxiety, introversion, disgruntlement), and impacts on emotional, social, and femininity roles (31), which can ultimately affect physical, mental, emotional, cognitive, and social aspects of women's quality of life (29, 42). PCOS can also have a negative impact on health-related quality of life in adolescents, and in these individuals, being overweight, hirsutism, acne, menstrual irregularities, and infertility have the most significant impact on the quality of life. These teenagers are more likely to express feeling less attractive and feminine than their peers. The disease also imposes psychosocial challenges in teenagers, so adolescents with PCOS may be more hostile and irritable than others. The effects of body weight, body mass index, and hirsutism in this age group need further evaluation (37).

### Theme 7: The effect of PCOS on sexual function

Theme 7 discusses the impact of the disease, treatment and complications on women's sexual function. The data from one study indicated that the frequency of sexual dysfunction in women with PCOS was 57.7%. Body mass index had a significant effect on sexual desire and arousal, while the effect of hirsutism was significant in all areas studied except dysphoria. Sexual function screening in all women with PCOS with a simple, short questionnaire such as the female sexual function index might be appropriate (33). Also, in some studies, sexual function was lower in the domains of desire (48.3%) and arousal (7.44%). Women with low levels of education and irregular menstrual status have been shown to be more prone to sexual dysfunction (8, 33). Infertility and hair loss have more significant effects on women's sexual function than other complications of PCOS. Accordingly, in one study, women with PCOS and infertility had lower sexual function scores in all domains except for desire and pain, than those who were fertile (38).

### Theme 8: The effect of reproductive complications of PCOS on the concerns and psychological health of women with PCOS

Theme 8 involves the effects of reproductive complications of the disease such as menstrual problems, infertility, weight gain, emotional disorders, and excess body hair on the psychological aspects of women' lives. Women's concerns such as worries about physical symptoms, the economic burden of the disease, fear of future complications, and implications of chronic disease can be easily underestimated or ignored in the disease management process (41). Findings indicate that women with PCOS face daily challenges; these include physical challenges (e.g., feeling unhealthy), social challenges (e.g., feeling old and disabled), and emotional challenges (e.g., worrying about aging with a chronic illness). Therefore, there is a need for comprehensive health care and psychological support for the treatment of PCOS in adolescents and young women in the early decades of reproductive ages (36). Women need specific support from health care workers to reduce anxiety and improve disease management by changing their lifestyle (43). Designing a comprehensive health program can improve women's mental health. One study also showed that a mental health program for women with PCOS offered by a multidisciplinary team was able to improve fertility outcomes while also being cost-effective (30).

### Theme 9: The effect of PCOS on the role of femininity

Theme 9 discusses the negative impact of the disease on women's social roles. The results showed that women's lives are strongly influenced by the norms of femininity in society. Some women with POCS may consider their body as `different' because of the PCOS symptoms, i.e., hirsutism. They may use different strategies to achieve body ideals and cope with symptoms. Hirsutism can have a decisive negative impact on women's daily lives, especially in relation to male partners and sexual relations (26). The symptoms of PCOS can also hinder the social role of women as parents. The results of one study showed that women's personal experience with PCOS were often overlooked (23).

## 4. Discussion

In this study, from 27 selected articles, 9 main themes on SRH aspects in women with PCOS were extracted. The World Health Organization defines SRH services and aspects as follows: prevention, diagnosis, care, and treatment services related to prenatal care, delivery and childbirth, family planning services including those concerning infertility and contraception, abortions, STIs, HIV/AIDS, cervical cancer, and sexual health (44). These SRH aspects are relevant for the general public regardless of their medical condition.

According to the present study's findings, women with PCOS have both general and specific needs for SRH. Women with PCOS may have fertility complications such as menstrual irregularities, infertility, and abnormal uterine bleeding that can affect their health throughout their lives. Because of these complications, these women need to be aware of their appropriate fertility and health preferences according to their circumstances in order to make the right health decisions. Also, the disease's financial burden can be high, particularly in cases of infertility and menstrual irregularities. The disease can have physical, mental, emotional, cognitive, and social effects that can reduce the quality of life of women. It can also affect the sexual health of women. Women also report concerns about these physical symptoms, the economic burden of the disease, fear of future complications, and illnesses that generally affect their health.

Considering the prevalence of this disease, prevention is important and it is recommended that health officials provide preventative programs such as disease screening programs for women and girls with obesity and hirsutism (32). One study reported that globally 1 in 5 women of childbearing age with obesity and hirsutism is affected by PCOS, and that the disease has clinically significant consequences for fertility, including infertility, hyperandrogenism, and hirsutism (19). Menstrual disorders and infertility have direct effects on reproductive health. Pregnancy and its related care are often affected by infertility and its consequences. Menstrual disorders can also affect ovulation, pregnancy, and contraception (34).

One study showed that PCOS had profound effects on the pathophysiology and clinical manifestations of reproductive function through a variety of mechanisms that led to androgen excess, increased free androgen availability and alterations of granulosa cell function and follicle development (35). These mechanisms involve early hormonal and metabolic factors during intrauterine life, leptin, insulin and the insulin growth factor system, and potentially the endocannabinoid system. Eventually, this trend can lead to women with PCOS, especially those who are obese, being characterized by worsened hyperandrogenic and metabolic status, and poor menses and ovulatory performance, ultimately leading to poor pregnancy rates (45).

The findings of the included studies showed that this disease can affect women's reproductive health from fetal life through adulthood and menopause. In this regard, PCOS can affect the levels of sex hormones, the menstrual cycle and ovulation status, and cause fertility disorders (21). The disease can start from the fetal period with symptoms of hyperandrogenism and insulin resistance, which can lead to numerous health problems in adolescence, adulthood, and old age. Menstrual and fertility problems will be replaced by metabolic complications with aging.

Medications and other therapeutic approaches are often used to treat the most common manifestations in each age group, such as irregular menstruation and hair growth during puberty, reproductive problems in adulthood, and metabolic issues and cancer risk in old ages. Careful monitoring at every life stage is essential to avoid health risks that may affect children, as risk of fetal and postpartum complications appear to be higher in women with PCOS (6).

One study suggested that although PCOS may have some genetic components, the clinical features of this disorder may change throughout the life stages, and lifelong evaluation of PCOS is necessary. In young women with PCOS, hyperandrogenism, menses irregularities, and insulin resistance represent the pathophysiological role of excess androgen and insulin in PCOS. Hyperandrogenism and infertility are important issues related to PCOS in reproductive ages. It has been found that, later in life, women with PCOS and obesity (particularly those with abdominal obesity) are more susceptible to developing type 2 diabetes mellitus (19). Little is known about ovarian morphology and androgen production in postmenopausal women with PCOS; some studies have shown that polycystic ovaries are prevalent in them, which are associated with high testosterone levels and metabolic changes. Identifying the major features of PCOS at different ages, is necessary to plan individual therapeutic strategies and possibly prevent chronic metabolic complications (45). Therefore, in planning to provide SRH services and considering the overall complications of the disease, specific problems should be considered at every stage of a woman's life.

This study showed adverse pregnancy outcomes in women with PCOS such as gestational diabetes, preeclampsia, preterm labor, fetal death, neonatal death, low Apgar scores, meconium aspiration, macrosomia, and large for gestational age and small for gestational age newborns (22). These complications can be reduced by providing appropriate prenatal care, care during and after delivery, and treatment of obstetric complications. One study showed that PCOS was associated with maternal and neonatal complications such as: miscarriage, hypertensive disorders, gestational diabetes, and preterm labor; and also higher risk of developing chronic illnesses in the women and their offspring (46).

This review highlights the educational needs of reproductive-aged women with PCOS about fertility, pregnancy, birth type, and health service preferences. There are limited studies about the reproductive health needs of women with PCOS, and their knowledge about the complications and treatment of this disease. Addressing these needs, informing the women about childbirth decisions, and identifying health priorities are essential for women with PCOS (40, 47). Thus, incorporating educational programs in the management of PCOS is very important.

The results of the present study showed that women with chronic diseases such as PCOS are concerned about the birth of their baby and have questions about the potential consequences of childbearing. Moreover, women with PCOS consider information necessary. With information about the disease, affected women can decide on treatment strategies and lifestyle choices and participate in joint decision-making with physicians (47).

According to the results of this study, because of the financial burden of PCOS, screening for this syndrome and preventing its complications seem to be a financially cost-effective strategy. This should be considered in health planning by policymakers. The results from one study demonstrated that the total cost of assessing and caring for reproductive-aged women with PCOS in the United States was 4.36 billion dollars in 2005 (24). As the cost of diagnosing PCOS is just a small part of the total costs of the disease (approximately 2%), general screening may be a cost-effective strategy, because it could lead to early diagnosis and intervention, and thus potentially health improvement and prevention of serious consequences from POCS (24). The financial burden of each complication of the disease should be assessed according to the affected woman's age.

The results of this study also showed sexual disorders in PCOS. PCOS can impair a women's sexual function due to complications such as ovulation disorders, obesity, and hyperandrogenism. A study showed that PCOS had significant effects on sexual desire and stimulation, while hirsutism affected all sexual domains that were examined except dyspareunia (8). PCOS can adversely affect women's sexual function in domains of desire, stimulation, lubrication, orgasm, satisfaction, and pain. Studies have also shown that among the symptoms of PCOS, infertility, and hair loss have more negative effects on sexual function (48). Thus, it is essential for health care providers to evaluate women's sexual function and educate them about sexual problems to promote women's sexual health.

This study also found evidence for psychological concerns in women with PCOS. The most common psychological issues were fears about physical symptoms, the financial burden of the disease, future complications, and chronic illnesses. The most common worries were about being overweight, menstrual problems, infertility, emotional problems, and hirsutism. Most psychological consequences of the disease are underestimated and overlooked by health service providers (49). Therefore, women need to receive special care to reduce their anxiety and improve their disease management (43).

Women with PCOS are also more susceptible to mood disorders and depression (41). Studies have suggested that PCOS can be associated with psychiatric disorders, and it is recommended to consider these disorders in the management of PCOS (50, 51). Therefore, after PCOS diagnosis, it is essential to screen for psychological disorders and refer to appropriate counseling centers, if needed (52).

Also, this study looked at the role of femininity in women with PCOS. It was observed that PCOS symptoms can negatively affect patients' roles and responsibilities as women and mothers, and reduce their public activities (50). The most important issue for women with PCOS is treating the physical complications, and usually their social roles and femininity are overlooked. Therefore, in assessing SRH needs, it is imperative to consider feelings of femininity and perceptions of roles of these women.

## 5. Conclusion

In many health systems designed to provide SRH services, women with PCOS are not given special attention, and their needs are assessed similarly as women without PCOS.

In the present study, it was shown that the complications of this disease affect SRH aspects of women throughout their lives from the embryonic stage to old age. Complications of pregnancy and childbirth can include preeclampsia, preterm delivery, gestational diabetes, and neonatal complications (20). The disease can also have adverse effects on women's sexual functions. Some studies have shown effects of complications such as hirsutism, obesity, skin and hair problems, and infertility on sexual function. Therefore, women with PCOS have different SRH needs, which should be specifically considered in the service delivery system (26).

PCOS and its complications cause a high financial burden that is often ignored in women's SRH needs. Thus, more research is needed to assess the costs in different countries. Also, PCOS can affect women's mental status; the psychological complications are often hidden and ignored. Because of the life-long nature of PCOS, affected women may need different care compared to the general population in each stage of their lives, and health systems should provide tailored services to these women (20, 49). To meet the SRH needs of women with PCOS, the researchers of this study suggest that health services for these women should be presented as a complete package of treatment based on the actual needs of the women.

##  Conflict of interest

The authors declare that there is no conflict of interest.
